# Implementation of ORBEYE^®^-Exoscope in the Operative Treatment of Spinal Dural Arteriovenous Fistula

**DOI:** 10.3390/medicina61010101

**Published:** 2025-01-11

**Authors:** Nikolay Tonchev, Belal Neyazi, Klaus-Peter Stein, I. Erol Sandalcioglu, Ali Rashidi

**Affiliations:** Department of Neurosurgery, University Hospital of Magdeburg, 39120 Magdeburg, Germany; belal.neyazi@med.ovgu.de (B.N.); klaus-peter.stein@med.ovgu.de (K.-P.S.); erol.sandalcioglu@med.ovgu.de (I.E.S.); ali.rashidi@med.ovgu.de (A.R.)

**Keywords:** Orbeye^®^, exoscope, neurosurgery, spinal dural arteriovenous fistula

## Abstract

Spinal dural arteriovenous fistulas (sDAVFs) are rather uncommon lesions of the spine. In sDAVFs, which represent the most frequent form of vascular malformations of the spine, operative treatment remains the most common treatment modality. In operative surgery, visualization and pathology detection have a key impact on the results of the neurosurgical treatment of an sDAVF. The exoscope is one of the most recent imaging devices developed alongside the microscope and endoscope. The exoscope is being increasingly applied in neurosurgical procedures as an alternative to operative microscopes due to various advantages, such as its non-space-occupying camera, excellent visualization of the anatomical details and the perfect teaching possibilities it thus provides. In this publication, we present our experience in the treatment of a patient with an sDAVF, where surgery was performed exclusively with an ORBEYE-exoscope for the entire duration of the procedure. This report outlines the workflow and some of the technical pitfalls involved in managing this vascular pathology using the exoscopic technique.

## 1. Introduction

Spinal dural arteriovenous fistulas (sDAVFs) represent an extremely rare vascular pathology of the spine [1,2,3,4]. Their classification is based on pathophysiology, pathoanatomy and diagnostic imaging [3,5,6]. The localization of an sDAVF is most frequently in the lower thoracic spine and lumbar region [7]. Arterialization of the medullary vein and increased venous pressure lead to perimedullar venous congestion, ischemia and in some cases hemorrhage [3,5,6,8,9,10].

The clinical symptoms are usually nonspecific, which is why the diagnosis occurs fairly late. At the time of presentation, many patients already suffer from severe neurological deficits of the lower extremities [11,12,13].

Early diagnosis to prevent the progression of symptoms, a spinal cord ischemia or infarction and initiating therapy are mandatory [11]. Magnetic resonance imaging (MRI) demonstrates specific morphological changes in the spinal cord with edema and perimedullar varicosis [14,15] and spinal digital subtraction angiography (DSA) can precisely visualize the level and site of feeding artery, fistula and draining venous system [11]. Treatment options include surgical occlusion of the intradural arterialized vein and endovascular therapy with a liquid embolic after super-selective catheterization of the feeding radiculomeningeal artery [7]. Nevertheless, microsurgical treatment remains the gold-standard modality because of its significantly lower failure rate (5% vs. 46%, *p* < 0.01) and lower rate of persistent neurological complications [16,17].

Intraoperative visualization of the sDAVF plays a crucial role in the success of neurosurgical treatment. In high-volume neurosurgical centers, the combination of microsurgery and intraoperative DSA gives promising results for reducing the error obliteration rate to zero percent [18]. However, hybrid ORs are not available to all specialists. As an alternative to this rather expensive technology, intraoperative visualization can be performed using a conventional surgical microscope with ICG functions. An emerging alternative to the classical intraoperative microscope (IOM) is exoscope-assisted surgery. Using high-definition digital camera systems, exoscope devices are designed to deliver real-time high-definition images of the surgical field to an external high-resolution monitor. This eliminates the necessity to look directly through oculars and allows for a greater freedom of movement during surgery. The latest technological advancements have also made the integration of 4K-3D technology, multiscreen outputs and intraoperative ICG video angiography into exoscope devices possible. We present our first experience in the treatment of a patient with an sDAVF visualized with the exoscope (ORBEYE^®^, Olympus-International, Crewe, UK), highlighting general aspects and technical considerations compared to conventional surgical microscopes.

## 2. Detailed Case Description

**Figure 1:**
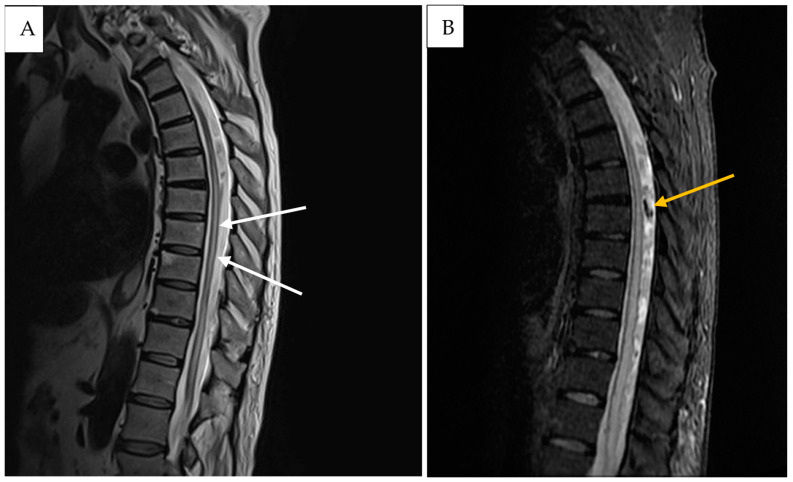
Case illustration: Pre-operative sagittal T2-weighted (**A**) and FLAIR MR imaging (**B**) of the thoracic region shows diffuse central spinal cord edema (white arrows) and flow voids (yellow arrow).

**Figure 2:**
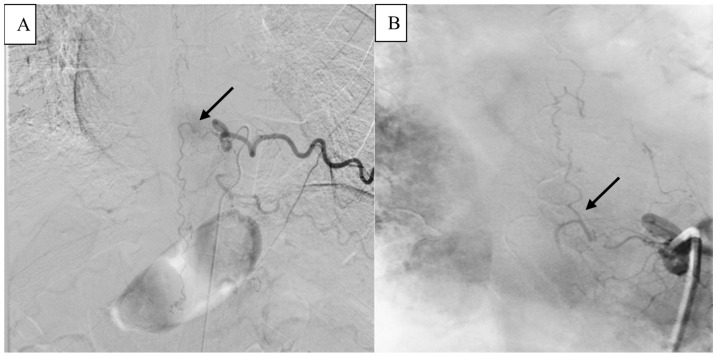
(**A**) View of super-selective digital subtraction angiography (DSA) with the spinal DAVF showing the feeders from the radicular meningeal artery. (**B**) After the shunt spot (black arrows), the AV-Fistula continues upwards intradurally, draining into the perimedulary veins.

A 72-year-old patient presented with a new onset of abnormal gait 4 months after a fall from a ladder. Due to the unclear clinical picture, the patient was referred by his family physician to a neurologist. The initial neurological examination revealed a slight spastic gait disturbance with sensory loss from T11 downwards. Because of the symptoms, the patient underwent a standard MRI of the spine, which showed only some nonspecific signal changes in the lower thoracic region. An electrophysiological exam of the lower extremities revealed additional slight axonal polyneuropathy, which did not explain the clinical manifestation. Initial clinical symptoms deteriorated during the next 5 months and the patient developed a progressive inability to walk normally and a new onset of bladder dysfunction. Due to the deterioration of their symptoms, the patient was referred to our university hospital center. The detailed neurological examination revealed reduced muscle strength for both legs (right leg 4/5, left leg 3/5) together with hyperreflexia in the patellar tendon reflex and achilles tendon reflex on both sides. A repeated MRI of the spinal axis depicted a progressive myelopathy, dilated perimedullary vessels and venous stasis (Figure 1). A subsequent spinal DSA proved the diagnosis of sDAVF at the spinal thoracic level, T9–10 (Figure 2).

**Figure 3:**
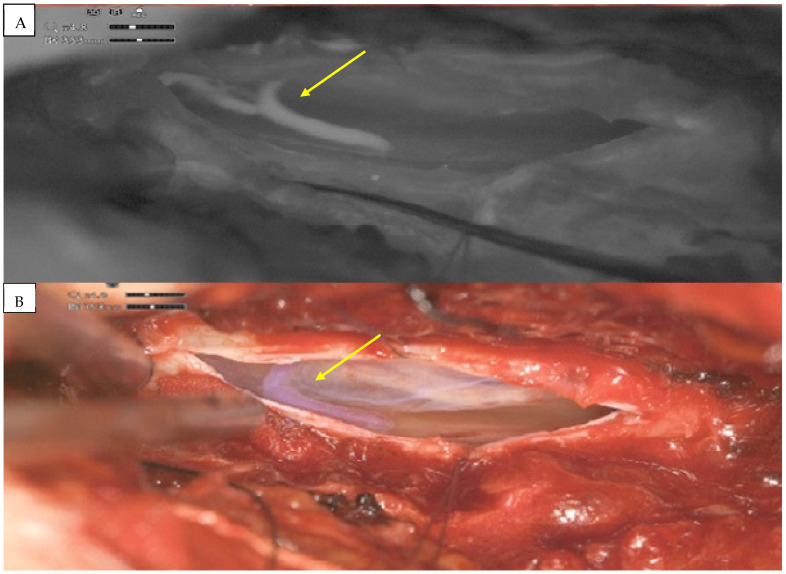
Spinal thoracic (T9) DAVF seen in ICG video-angiography mode (**A**) using digital 3D exoscope. Intraoperative ORBEYE-exoscope view (**B**) of the arterialized intradural vein (yellow arrows).

**Figure 4:**
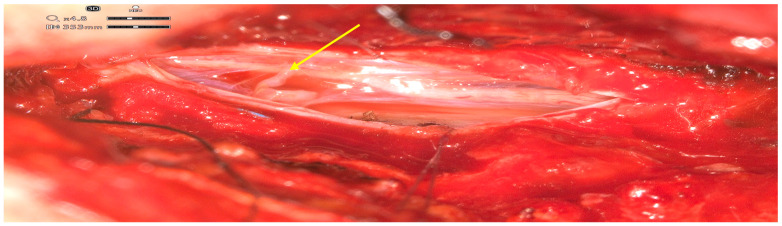
High-definition intraoperative exoscope image of the intradural venous vessels (yellow arrow) after bipolar coagulation.

**Figure 5:**
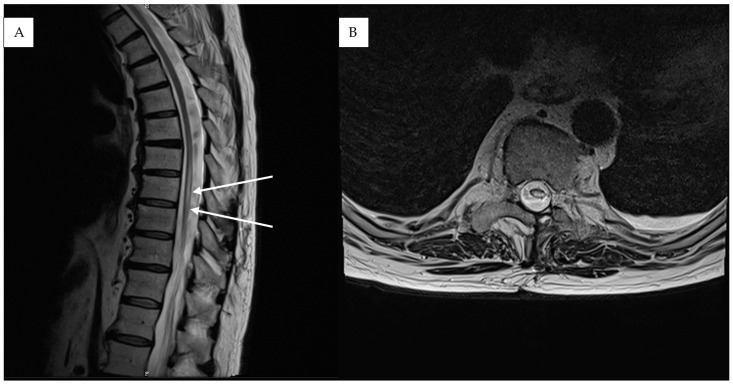
Case results: Post-operative sagittal (**A**) and axial (**B**) T2-weighted MR images show decrease in spinal cord edema (white arrows) three months after the operation.

After discussions amongst the interdisciplinary vascular board, surgical occlusion was recommended. A complete surgical procedure was performed with the ORBEYE-exoscope. A hemilaminectomy at the Th9–Th10 level was performed and the spinal dura was exposed. After the dural opening, the fistula point and arterialized vein were, respectively, exposed. Pathological vessels were visualized using exoscope-integrated fluorescence angiography with indocyanine-green (ICG) (Figure 3). Switching between normal light to ICG mode was comfortable and the quality of imaging was, in our opinion, equivalent or even higher than that using standard microscope modules. The sDAVF was occluded by coagulation (Figure 4) and then resected. After dural closure, further wound closure was performed in a typical manner. The complete elimination of the sDAVF was confirmed by postoperative digital subtraction angiography (DSA) on the third postoperative day. The patient did not experience any neurological deterioration following surgery and was discharged home in a stable clinical condition 10 days after the operation. Three months later, the neurological symptoms revealed partial recovery. A postoperative MRI at the 3 month follow-up showed regressive spinal cord edema with still-detectable thoracolumbar myelopathy (Figure 5).

## 3. Results

We compared our previous experience with the OM to the exoscopy setting. The transition from a conventional surgical microscope to the exoscope influenced the entire operation team and offered several advantages. First and foremost, the position of the exoscope offered more working space and easier instrumentation. At the same time, the quality of the screen images in terms of contrast and brightness was at least as good as that of the classic operating microscope. One of the features of the exoscope provides integrated digital light source calibration, which enables a fluid workflow. ORBEYE provided a very satisfactory performance in terms of the depth of focus and resolution.

## 4. Discussion

Spinal dural arteriovenous fistulas are rare pathologies, which can lead to progressive myelopathy. Early detection is mandatory, because neurological deficits are potentially reversible depending on the severity and duration of symptoms [11]. Typical MRI characteristics are hyperintense centromedullary edema on T2-weighted images, and the presence of flow voids, which represent dilated perimedullary vessels [3]. Surgical occlusion and endovascular embolization of these lesions are the treatment options. Surgical therapy has been shown to be up to 98% successful, but endovascular embolization provides less favorable results for this type of fistula [3]. Several pitfalls become relevant when planning the microsurgical treatment of spinal DAVFs. One of the main concerns is precise level localization. In the thoracic region, this can be sometimes difficult even in terms of using intraoperative C-arm fluoroscopy. Another possible source of failure can be the difficulty of visualizing major and minor feeders in cases of multiple feeders. Because hybrid operating rooms with high-quality angiography are not always available, additional options such as intraoperative ICG angiography become crucial in terms of the microsurgical treatment of sDAVFs. We present our surgical experience in the treatment of an sDAVF using the ORBEYE^®^-exoscope and discuss the technical benefits of this technique.

The main feature of the 3D exoscope is its use as a teaching tool. Other benefits of the exoscope compared to operative microscope (OM) as an intraoperative visualization tool in neurosurgical procedures include improved visualization of the surgical field and ergonomics [19]. During the aforementioned surgical procedure, there was no need for lateral tilting of the surgeon’s upper body when addressing the cranial and caudal parts of the operating field, as would have been needed in case of OM usage. Additionally to this, using the exoscope allowed us to perform even small adjustments to the view without the need to remove the surgeon’s hands from the surgical field and interrupt the micromanipulation of the intradural vessels. We experienced a benefit from using additional illumination from a theater lamp. In terms of the steepness of the learning curve, there are diverse results in the literature, with a steep learning curve reported for microvascular anastomosis with the exoscope, which has not been achieved as well as with OM [19,20]. One single-center study by Ariffin et al. [21] reported a rather short learning curve and adaptation to the exoscope. These results comply with the findings from the retrospective data-review by Kwan et al., who experienced excellent surgical and clinical outcomes without any complications during the implementation of an exoscope in spinal surgery [22]. However, more experienced neurosurgeons tend to experience difficulties during the transition from an OM to an exoscope mostly because of previous motor schemes obtained through the use of operating microscopes.

One of the main advantages of the exoscope is that it enables all staff present in the operating room to have the same quality of visualization of the surgical field, which in the past was only possible for surgeons under OMs [19,22,23]. This way, the assistant as well as other observers in the surgical hall are exposed to the same intraoperative view as the main surgeon. The integration of robotics in exoscope-assisted spine surgery facilitates neurosurgeons in navigating challenging visualization trajectories. The exoscope, in contrast to the standard OM, is characterized by its compact size, which confers certain benefits during spine surgery procedures. These benefits become particularly apparent in cases involving cervical soft tissue dissection, neural structure decompression and intradural preparation, where the freedom of hands’ movement can be crucial for the surgical outcome.

As pointed out by Montemurro et al., the complication rate with the use of exoscopes in spine surgery is not higher than that in OM surgery [24]. Similar results with no major additional risk to the patient during exoscope-assisted surgery for spinal DAVFs were reported by Auruccio et al. in a very recent publication [25]. This case series compared several surgical parameters such as blood loss and procedure time as well as treatment results between exoscope and microscope procedures. The results revealed a shorter operative time (141 vs. 151 min) and less blood loss (60 vs. 100 mL) in favor of surgical treatment using a digital 3D exoscope for spinal DAVFs.

Another positive aspect of the system is the possibility for the entire team to follow the surgery on the 3D-monitors, which also allows for stronger participation from the entire surgical team. In hospitals where no hybrid operating room is available, direct intraoperative visualization of the vascular architecture with an exoscope has the potential to eliminate the necessity for invasive studies such as post-procedural DSA. Future prospective studies are required to assess whether the implementation of an exoscope in the treatment for vascular spinal pathology could result in a reduction in the length of hospitalization and cost savings associated with this. Additionally, the integration of neuronavigation and artificial intelligence models could allow for more selective tissue recognition and an even wider range of implementation. Through continuous technological development and greater acceptance from neurosurgeons, the exoscope has the potential to refine clinical outcomes and reduce complications in the field of spine surgery.

While the exoscope technique is gaining popularity in the field of spinal surgery, there are still ongoing discussions about its efficacy in depth perception. Inefficient illumination in cases of deep and narrow surgical corridors makes this technique inferior in comparison to the standard OM. One important limitation of the current literature on exoscope use is the absence of long-term follow-up data. This restricts the precise evaluation of persistent benefits and potential complications. Further studies regarding the learning curve associated with transitioning to exoscope-assisted techniques are strongly recommended.

## 5. Conclusions

In our experience, the treatment of an sDAVF with an exoscope is a safe procedure with limited surgical risk and several advantages over classical OM surgery. The visual properties of ORBEYE allow for a more comfortable working position and facilitate team communication during surgery. With the ORBEYE-exoscope, we were able to safely resect an sDAVF. In general, this technique was adopted very quickly and did not show any disadvantages in comparison to the conventional OM setting.

## Data Availability

The datasets obtained and analyzed during the current study are available from the corresponding author on reasonable request.
